# Kidney function loss and albuminuria progression with GLP-1 receptor agonists versus basal insulin in patients with type 2 diabetes: real-world evidence

**DOI:** 10.1186/s12933-023-01829-0

**Published:** 2023-05-27

**Authors:** Meir Schechter, Cheli Melzer Cohen, Alisa Fishkin, Aliza Rozenberg, Ilan Yanuv, Dvora R. Sehtman-Shachar, Gabriel Chodick, Alice Clark, Trine J. Abrahamsen, Jack Lawson, Avraham Karasik, Ofri Mosenzon

**Affiliations:** 1 Diabetes Unit, Department of Endocrinology and Metabolism, Hadassah Medical Center, P.O.B 12000, 9112001 Jerusalem, Israel; 2 Faculty of Medicine, Hebrew University of Jerusalem, Jerusalem, Israel; 3 Department of Clinical Pharmacy and Pharmacology, University Medical Center Groningen, University of Groningen, Groningen, The Netherlands; 4 Maccabi Institute for Research and Innovation, Maccabi Healthcare Services, Tel-Aviv, Israel; 5 School of Public Health Sackler, Faculty of Medicine, Tel Aviv University, Tel Aviv, Israel; 6 Novo Nordisk A/S, Copenhagen, Denmark; 7 Tel Aviv University, Tel Aviv, Israel

**Keywords:** GLP-1 RA, Basal insulin, Chronic kidney disease, Real world evidence, Type 2 diabetes, eGFR slope, Albuminuria, Real world

## Abstract

**Background:**

In clinical trials enrolling patients with type 2 diabetes (T2D) at high cardiovascular risk, many glucagon-like peptide-1 receptor agonists (GLP-1 RAs) improved albuminuria status and possibly mitigated kidney function loss. However, limited data are available regarding the effects of GLP-1 RAs on albuminuria status and kidney function in real-world settings, including populations with a lower baseline cardiovascular and kidney risk. We assessed the association of GLP-1 RAs initiation with long-term kidney outcomes in the Maccabi Healthcare Services database, Israel.

**Methods:**

Adults with T2D treated with  ≥ 2 glucose-lowering agents who initiated GLP-1 RAs or basal insulin from 2010 to 2019 were propensity-score matched (1:1) and followed until October 2021 (intention-to-treat [ITT]). In an as-treated (AT) analysis, follow-up was also censored at study-drug discontinuation or comparator-initiation. We assessed the risk of a composite kidney outcome, including confirmed  ≥ 40% eGFR loss or end-stage kidney disease, and the risk of new macroalbuminuria. Treatment-effect on eGFR slopes was assessed by fitting a linear regression model per patient, followed by a t-test to compare the slopes between the groups.

**Results:**

Each propensity-score matched group constituted 3424 patients, 45% women, 21% had a history of cardiovascular disease, and 13.9% were treated with sodium-glucose cotransporter-2 inhibitors at baseline. Mean eGFR was 90.6 mL/min/1.73 m^2^ (SD 19.3) and median UACR was 14.6 mg/g [IQR 0.0–54.7]. Medians follow-up were 81.1 months (ITT) and 22.3 months (AT). The hazard-ratios [95% CI] of the composite kidney outcome with GLP-1 RAs versus basal insulin were 0.96 [0.82–1.11] (p = 0.566) and 0.71 [0.54–0.95] (p = 0.020) in the ITT and AT analyses, respectively. The respective HRs for first new macroalbuminuria were 0.87 [0.75–0.997] and 0.80 [0.64–0.995]. The use of GLP-1 RA was associated with a less steep eGFR slope compared with basal insulin in the AT analysis (mean annual between-group difference of 0.42 mL/min/1.73 m^2^/year [95%CI 0.11–0.73]; p = 0.008).

**Conclusion:**

Initiation of GLP-1 RAs in a real-world setting is associated with a reduced risk of albuminuria progression and possible mitigation of kidney function loss in patients with T2D and mostly preserved kidney function.

**Supplementary Information:**

The online version contains supplementary material available at 10.1186/s12933-023-01829-0.

## Introduction

Around 20–40% of patients with type 2 diabetes have chronic kidney disease (CKD), defined by either reduced kidney function, the presence of albuminuria, or other evidence of kidney damage [1–4]. CKD is associated with an increased risk of developing end-stage kidney disease (ESKD), cardiovascular complications, and mortality [5, 6]. Several classes of medications improve cardiovascular and kidney outcomes in patients with type 2 diabetes and CKD, including angiotensin-converting enzyme inhibitors (ACEi) [7], angiotensin receptor blockers (ARBs) [8], sodium-glucose cotransporter-2 inhibitors (SGLT2i) [9], and the non-steroidal mineralocorticoid receptor antagonist (MRA) finerenone [10, 11]. However, there is little evidence for therapies preventing kidney disease onset.

Many glucagon-like peptide-1 receptor agonists (GLP-1 RAs) improve cardiovascular outcomes [12] and albuminuria-based kidney endpoints [13–15] in patients with type 2 diabetes at high cardiovascular risk. Some evidence also suggests that GLP-1 RAs mitigate kidney function loss, especially in patients with evidence of kidney disease [12, 16], and this subject is being formally tested in the FLOW trial [17]. However, limited data are available regarding the kidney effects of GLP-1 RA in real-world settings [18], especially regarding albuminuria-based outcomes or eGFR slopes. Thus, it is unclear whether the findings from the clinical trials are generalizable to broader populations with type 2 diabetes.

In this observational cohort study, we used the Maccabi Healthcare Services (MHS) database, Israel's second-largest healthcare maintenance organization (HMO), to compare kidney outcomes in patients with type 2 diabetes initiating GLP-1 RAs versus basal insulin. We also assessed the risk for albuminuria progression and change in eGFR over time between treatment groups.

## Methods

### Study design, participants, and follow-up definitions

The MHS database includes around 180,000 patients with type 2 diabetes among its over 2 million registrees. We included adults with type 2 diabetes who initiated a GLP-1 RA (exenatide [introduced in Israel in 12.2007], liraglutide [2.2010], lixisenatide [1.2015], exenatide extended-release [11.2015], dulaglutide [4.2016], and semaglutide [8.2019]) or basal insulin between February 2010 to December 2019. The day of drug initiation was defined as the index date, and the year preceding the index date was defined as the baseline period. We selected basal insulin as a comparator to ensure comparability between the groups, because during these years in Israel both drugs were mainly used as injectable drugs for glycemic control in advanced stages of diabetes. Accordingly, we included patients treated with at least two other glucose-lowering agents (GLAs) at the baseline period, reflecting the common use at Israel at the time. Only those with at least one eGFR measurement at the baseline period were included. We excluded patients with type 1 diabetes, eGFR < 15 mL/min/1.73 m^2^, an indication of kidney transplantation or dialysis treatment, or those treated with the comparator drug within the year prior index date. Patients with an indication of pregnancy within 9 months before the index date were also excluded. To reduce bias associated with physicians' preference to treat severely ill patients with familiar and less costly drugs, we excluded patients with a diagnosis of dementia; history of organ transplantation; in MHS’ cancer (within the past 5 years) or heart failure registers; or those hospitalized for  ≥ 5 consecutive days within the past 180 days (Additional file 1: Figure S1).

In the protocol, we defined two follow-up periods. In the intention to treat (ITT) analysis, follow-up continued until the end of data availability, death, or October 2021. In the as-treated (AT) analysis, follow-up was censored also at exposure discontinuation (added by 180 days of grace period) or the initiation of the comparator. In addition, we performed a sensitivity analysis censoring the ITT follow-up for all patients after 4 years of follow-up (ITT-48mo). The rationale behind this analysis was to terminate follow-up when a large portion of the participants was still exposed to the study drugs. Four years cut-off was also selected to emulate the follow-up duration of the LEADER trial [19].

The study was approved by the institutional review board (IRB) at MHS. Due to the de-identified nature of the data, the IRB did not require obtaining informed consent from the participants.

### Definitions of baseline variables

Validated MHS registries were used to identify patients with type 2 diabetes, cardiovascular disease (including heart failure), hypertension, or cancer [20–22]. The relevant *International Classification of Diseases-9* diagnosis codes, *Anatomical Therapeutic Chemical* medications codes, and MHS registries are presented in Additional file 1: Table S1. Blood and urine samples included in this study were collected in community settings and were measured in the MHS-certified central laboratory. eGFR was calculated using the Chronic Kidney Disease Epidemiology Collaboration (CKD-EPI) equation [23]. Residential socioeconomic status (SES) was ranked on a 1 (lowest) to 10 (highest) scale. This score was derived by *Points Location Intelligence Ltd,* combining geographic and socioeconomic information for each neighborhood (e.g., expenditures related to retail chains, credit cards, and housing). This score is highly correlated with the SES measured by the Israeli Central Bureau of Statistics. This parameter was categorized into 4 groups (low [1–3], low-medium [4, 5], medium [6, 7] and high [8–10]) [24].

### Outcomes and subgroups

The main kidney outcome was a composite of confirmed  ≥ 40% eGFR reduction from baseline or new ESKD. Additional outcomes were confirmed or single-measurement eGFR reductions of  ≥ 30, ≥ 40, ≥ 50, or  ≥ 57% (corresponding to a doubling of serum creatinine) or new ESKD alone. We also assessed albuminuria outcomes: (1) a categorical increase of urine albumin-to-creatinine ratio (UACR) for the following categories:  < 30, 30- < 300 or  ≥ 300 mg/g; (2) or new-onset macroalbuminuria (UACR ≥ 300) among those with UACR ≤ 230 mg/g at baseline (resembling a ≥ 30% increase in UACR). The albuminuria-based outcomes were assessed either as a single- or as confirmed -measurement. In addition, we compared the eGFR slopes between the groups.

In addition to the entire study population, analyses were performed in subgroups defined by sex, age (< 60 or ≥ 60 years), presence of CVD, years in diabetes registry (≤ 10 or > 10 years), body mass index (< 30 or ≥ 30 kg/m^2^), HbA1c (< 8, or ≥ 8%), UACR (< 30, 30- < 300, or ≥ 300 mg/g), treatment with ACEi/ARBs, and treatment with SGLT2i. Patients were also divided into subgroups by their baseline eGFR (≥ 90 and < 90 mL/min/1.73 m^2^); this threshold was selected owing to the relatively preserved kidney function of the study population. As a sensitivity analysis, we also divided patients into three eGFR subgroups (≥ 90, 60- < 90, or < 60 mL/min/1.73 m^2^).

### Statistical analysis

Participants were propensity-score (PS) matched in 1:1 ratio using greedy matching, as previously described [25]. The model included 88 baseline parameters, including demographic variables (including SES), medical history, concomitant medications, and laboratory values (see the complete list in the Additional file 1). Continuous variables were categorized, and missing values were defined as a distinct ‘missing’ category to allow all patients to be matched. The PS matching was carried out by layers of baseline eGFR (> 90, 60–90, 45–60, 30–45, and 15–30 mL/min/1.73 m^2^).

Baseline values were described using mean and standard deviation (continuous variables with approximately normal distribution), median and IQR (continuous variables with skewed distribution), or proportions (categorical variables). Standardized difference (STD) was used to assess differences between the GLP-1 RAs and basal insulin group, with values of  < 10% considered negligible.

Cumulative incidence functions were used to describe the incidence of the outcomes in each group. Cox proportional hazard regression models were applied to estimate hazard ratios, confidence intervals, and p-value. The models were adjusted for the competing risk of mortality using cause-specific hazard models for the cumulative incidence functions and by using sub-distribution hazard functions for the Cox model [26].

The eGFR change from baseline at different time points was estimated using mixed models for repeated measures. We defined time windows of 6 months during the first 3 years and each year thereafter. At each time window, we included for each patient the eGFR measurement closest to the end of the period. Differences between groups at different time points were estimated using mixed-effect models with repeated measures. In randomized controlled trials (RCTs), eGFR slopes are also estimated using mixed-effect models with repeated measures; however, this approach may not fit the irregular sampling in real-world settings, where the times of measurements vary between patients. Therefore, to assess eGFR slopes, we fitted a linear regression model per patient (with time from index date as the independent variable and eGFR values as the dependent variable), enabling us to use all available eGFR measurements. The between-group difference in the linear slope estimates were compared using a t-test. We included in this analysis only patients with  ≥ 2 eGFR measurements with  ≥ 180 days between the first and last evaluations.

This study did not include formal hypothesis testing, and the p-values are presented for descriptive purposes only. No correction for multiple testing was performed. We consider a p < 0.05 as statistically significant. Analyses were performed using SAS version 9.4.

### Role of the funding sources

The study was funded by Novo Nordisk. The funder was involved in the study design, data analysis, data interpretation, writing of the report, and the decision to submit the paper for publication. This report was written according to a predefined protocol, including main and additional outcomes.

## Results

### Baseline characteristics

Overall, 11,634 and 22,598 patients initiated GLP-1 RAs or basal insulin, respectively. After applying the inclusion and exclusion criteria and PS-matching, there were 3424 participants in each group (Additional file 1: Figure S1). The mean age at baseline was 59.4 years (SD 9.4), and 44.8% were female. Participants at baseline had a mean diabetes duration of 9.7 years (4.6), body mass index of 33.4 (5.4) kg/m^2^, and HbA1c of 9.0% (1.4). Cardiovascular disease was prevalent in 21.0%, mean eGFR was 90.6 mL/min/1.73 m^2^ (19.3), and median [IQR] UACR was 14.6 mg/g [0.0–54.7]. At baseline, 74.9% were treated with ACEi/ARBs and 13.9% were treated with SGLT2 inhibitors. Baseline characteristics were well balanced between the groups following PS-matching. (Table 1 and Additional file 1: Tables S2 and S3).

**Table 1:** Baseline characteristics of initiators of GLP-1 RAs or basal insulin after propensity-score matching

Variable	Levels	GLP-1 RAs (n = 3424)	Basal insulin (n = 3424)	STD
Demographics				
Age (years)	Mean (SD)	59.5 (9.5)	59.3 (10.8)	0.02
Women (%)	n (%)	1530 (44.7)	1537 (44.9)	0.00
Socioeconomic status	1–3, n (%)	453 (13.2)	457 (13.3)	0.04
	4–5, n (%)	1060 (31.0)	1067 (31.2)	
	6–7, n (%)	1172 (34.2)	1217 (35.5)	
	8–10, n (%)	736 (21.5)	681 (19.9)	
	Missing, n (%)	3 (0.1%)	2 (0.1%)	
	Mean (SD)	5.9 (2.0)	5.8 (1.9)	0.04
Medical history				
Years in diabetes registry	Mean (SD)	9.8 (4.6)	9.6 (4.6)	0.04
Established CVD history*	n (%)	729 (21.3)	709 (20.7)	0.01
Hypertension registry*	n (%)	2390 (69.8)	2376 (69.4)	0.01
BMI kg/m^2^	Mean (SD)	33.6 (5.1)	33.3 (5.6)	0.05
	Missing, n (%)	125 (3.7%)	129 (3.8%)	
HbA1c (%)	Mean (SD)	9.0 (1.4)	9.0 (1.5)	−0.01
	Missing, n (%)	3 (0.1)	8 (0.2)	
Medications				
Metformin	n (%)	3316 (96.8)	3335 (97.4)	−0.03
Sulfonylureas 2nd generation	n (%)	1860 (54.3)	1858 (54.3)	0.00
SGLT2i	n (%)	475 (13.9)	475 (13.9)	0.00
RAAS inhibitors	n (%)	2582 (75.4)	2547 (74.4)	0.02
Thiazolidinediones	n (%)	233 (6.8)	247 (7.2)	−0.02
Fast acting insulin	n (%)	34 (1.0)	35 (1.0)	0.00
Beta blockers	n (%)	1320 (38.6)	1286 (37.6)	0.02
Aldosterone antagonists	n (%)	100 (2.9)	88 (2.6)	0.02
Antihypertensives	n (%)	2742 (80.1)	2729 (79.7)	0.01
Kidney markers				
eGFR (ml/min/1.73 m^2^)	> 90, n (%)	2080 (60.7)	2080 (60.7)	0.00
	60–90, n (%)	1046 (30.5)	1046 (30.5)	
	45–60, n (%)	219 (6.4)	219 (6.4)	
	30–45, n (%)	70 (2.0)	70 (2.0)	
	15–30, n (%)	9 (0.3)	9 (0.3)	
	Mean (SD)	90.1 (18.8)	91.2 (19.8)	−0.06
UACR (mg/g)	Below detectable, n (%)	1167 (34.1)	1211 (35.4)	0.04
	< 15, n (%)	448 (13.1)	426 (12.4)	
	15- < 30, n (%)	472 (13.8)	470 (13.7)	
	30- < 300, n (%)	864 (25.2)	874 (25.5)	
	≥ 300, n (%)	256 (7.5)	234 (6.8)	
	Missing, n (%)	217 (6.3)	209 (6.1)	
	Median (IQR), n	14.8 (0.0–55.0)	14.2 (0.0–54.7)	0.03

*BMI* body mass index, *CVD* cardiovascular disease, *eGFR* estimated glomerular filtration rate, *GLP-1 RA* glucagon-like peptide 1 receptor agonist, *RAAS* renin angiotensin aldosterone system, *SGLT2i* sodium-glucose transporter 2 inhibitors, *STD* standardized difference, *UACR* urine albumin-to-creatinine ratio

^*^Based on Maccabi Helathcare Services validated registries

### Main outcome overall and by subgroups

**Fig. 1:**
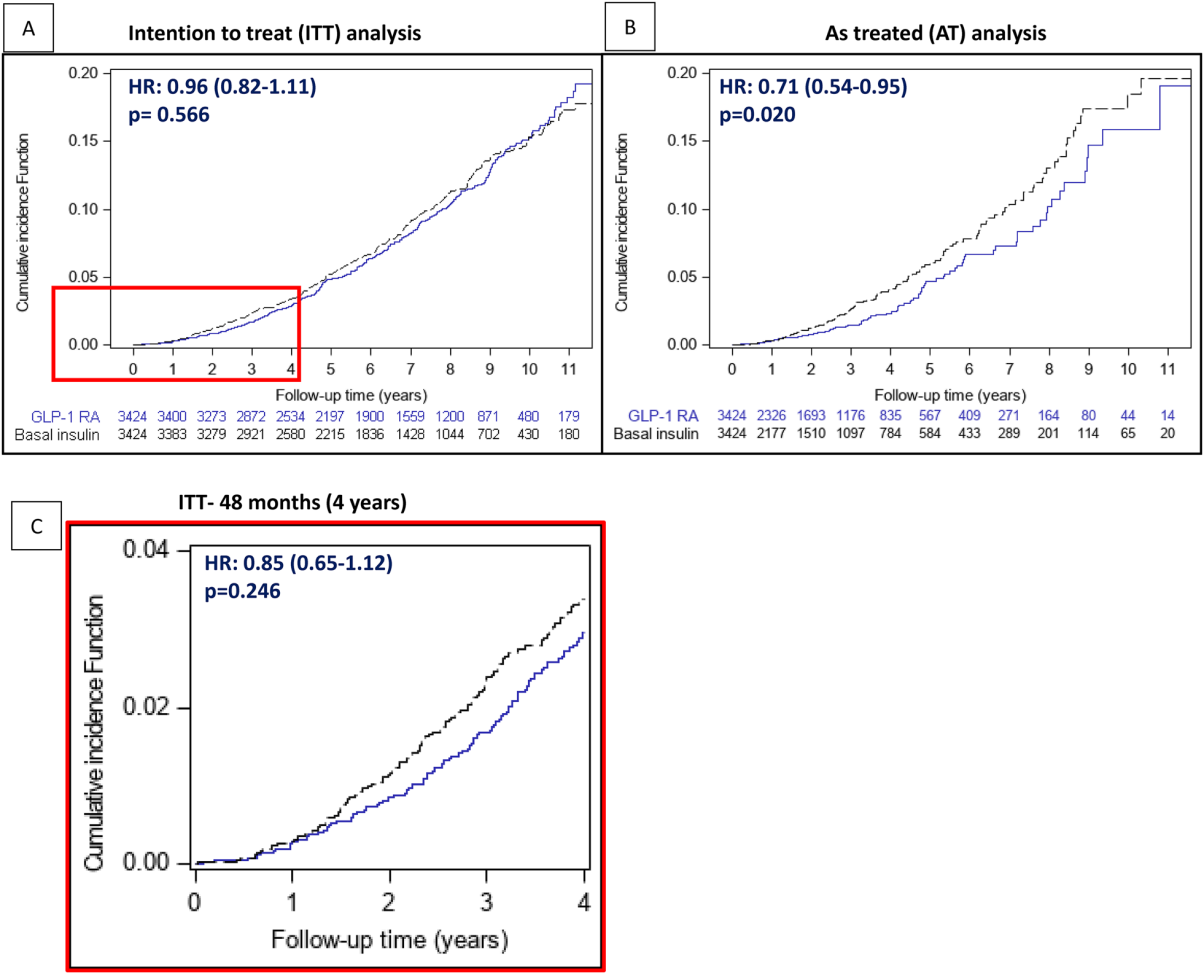
The association between initiation of GLP-1 RAs versus basal insulin and the risk of the composite kidney outcome. The risk of the composite kidney outcome (confirmed ≥ 40% eGFR decline or new end-stage kidney disease) was compared between initiators of GLP-1 RAs versus basal insulin. Presented are cumulating incidence functions. In an intention-to-treat (ITT) analysis, patients were followed until October 2021, the end of data availability, or death (**A**). In an as-treated (AT) analysis, follow-up was also censored at study drug discontinuation or comparator-initiation (**B**). In an ITT-48 months (ITT-48mo) analysis, to emulate the LEADER study, the ITT follow-up was also censored at four years when a large proportion of the participants were still on the study drugs (**C**). Cox proportional hazards regression models were applied to compare between treatment arms. *GLP-1 RA* Glucagon-like peptide-1 receptor agonists, *eGFR* estimated glomerular filtration rate

**Fig. 2:**
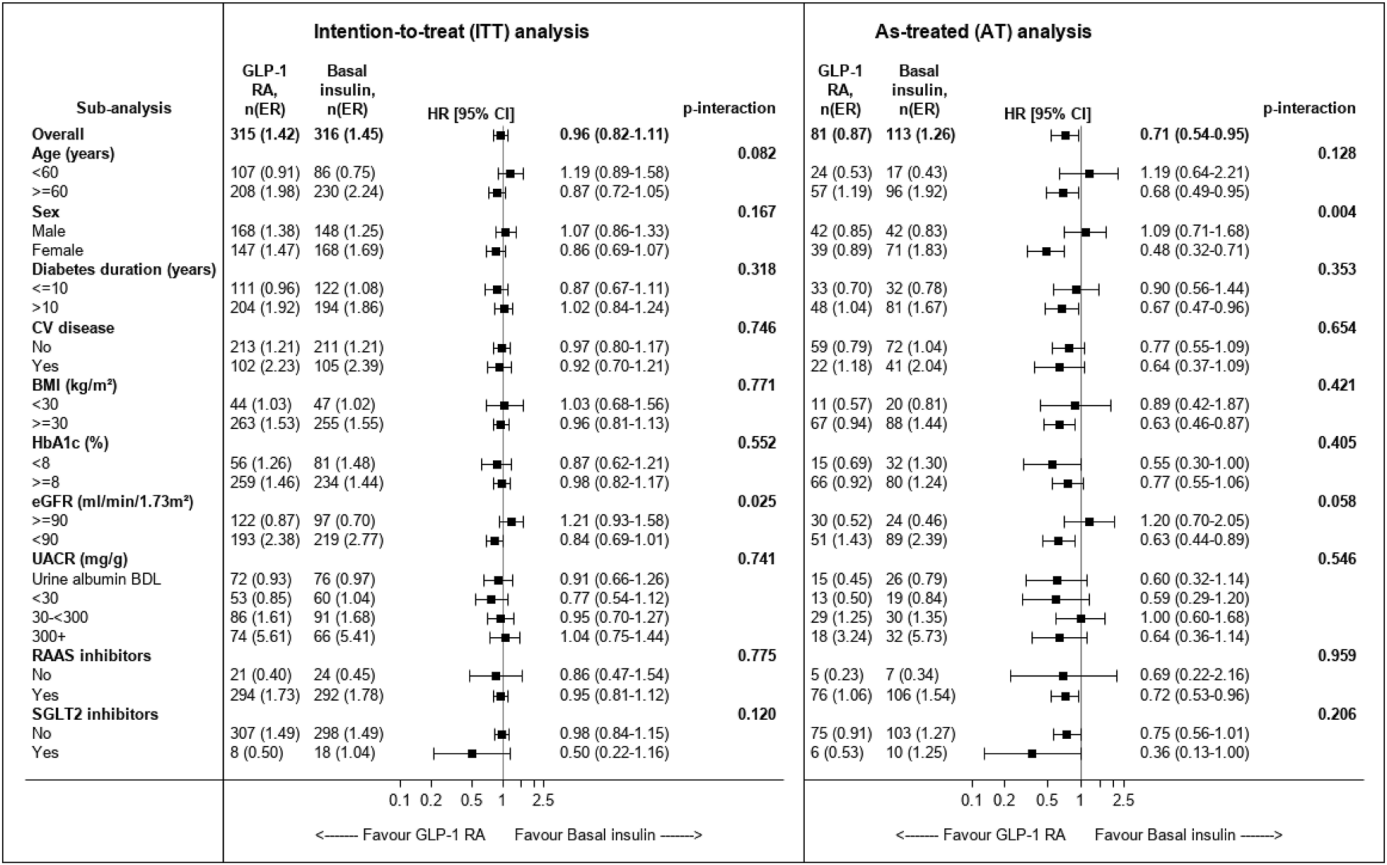
The association between initiation of GLP-1 RAs versus basal insulin and the risk of the composite kidney outcome by baseline subgroups. The risk of the composite kidney outcome (confirmed ≥ 40% eGFR decline or new end-stage kidney disease) was compared between initiators of GLP-1 RA and initiators of basal insulin across subgroups. In the intention-to-treat (ITT) analysis, patients were followed until October 2021, end of data availability or death. In the as-treated (AT) analysis, follow-up was also censored at study drug discontinuation or comparator-initiation. Cox proportional hazards regression models were applied to compare treatment arms, with an interaction term between subgroups and the treatment arm. Event rates (ER) are per 100 patients-years. *BDL* below detectable levels, *BMI* body mass index, *CV* cardiovascular, *GLP-1 RA* Glucagon-like peptide-1 receptor agonists, *HbA1c* glycated hemoglobin A1c, *eGFR* estimated glomerular filtration rate, *RAAS* Renin angiotensin aldosterone system, *SGLT2* sodium-glucose co-transporter 2 inhibitors, *UACR* urine albumin-to-creatinine ratio

In the ITT follow-up, during a median of 81.1 months [IQR 50.9–110.0], the median number of eGFR measurements per patient was 14 and 13 [7–22 and 7–20] for GLP-1 RAs and basal insulin group, respectively (Additional file 1: Tables S4 and S5). The composite kidney outcome (≥ 40% eGFR loss or ESKD) occurred overall in 631, 199, and 194 patients in the ITT, ITT-48mo, and AT follow-ups, respectively (Fig. 1 and Additional file 1: Figure S2). The hazard ratios of this outcome with GLP-1 RAs compared to basal insulin, were 0.96 ([95% CI 0.82–1.11]; p = 0.566), 0.85 ([0.64–1.12]; p = 0.246), and 0.71 ([0.54–0.95]; p = 0.020) in the ITT, ITT-48mo, and AT follow-ups, respectively (Fig. 1). There was no evidence that the effect varies across most tested subgroups, including by baseline treatment with SGLT2i (Fig. 2). The treatment effect seemed to be more pronounced in patients with eGFR < 90 compared to eGFR ≥ 90 mL/min/1.73 m^2^ (p-interaction = 0.025 [ITT] and 0.058 [AT]). This association was not significant when those with eGFR < 90 were further divided into eGFR < 60 or 60- < 90 mL/min/1.73 m^2^, although the direction was similar (p-interaction = 0.083 [ITT] and 0.129 [AT]) (Additional file 1: Table S6).

### Other study outcomes

**Fig. 3:**
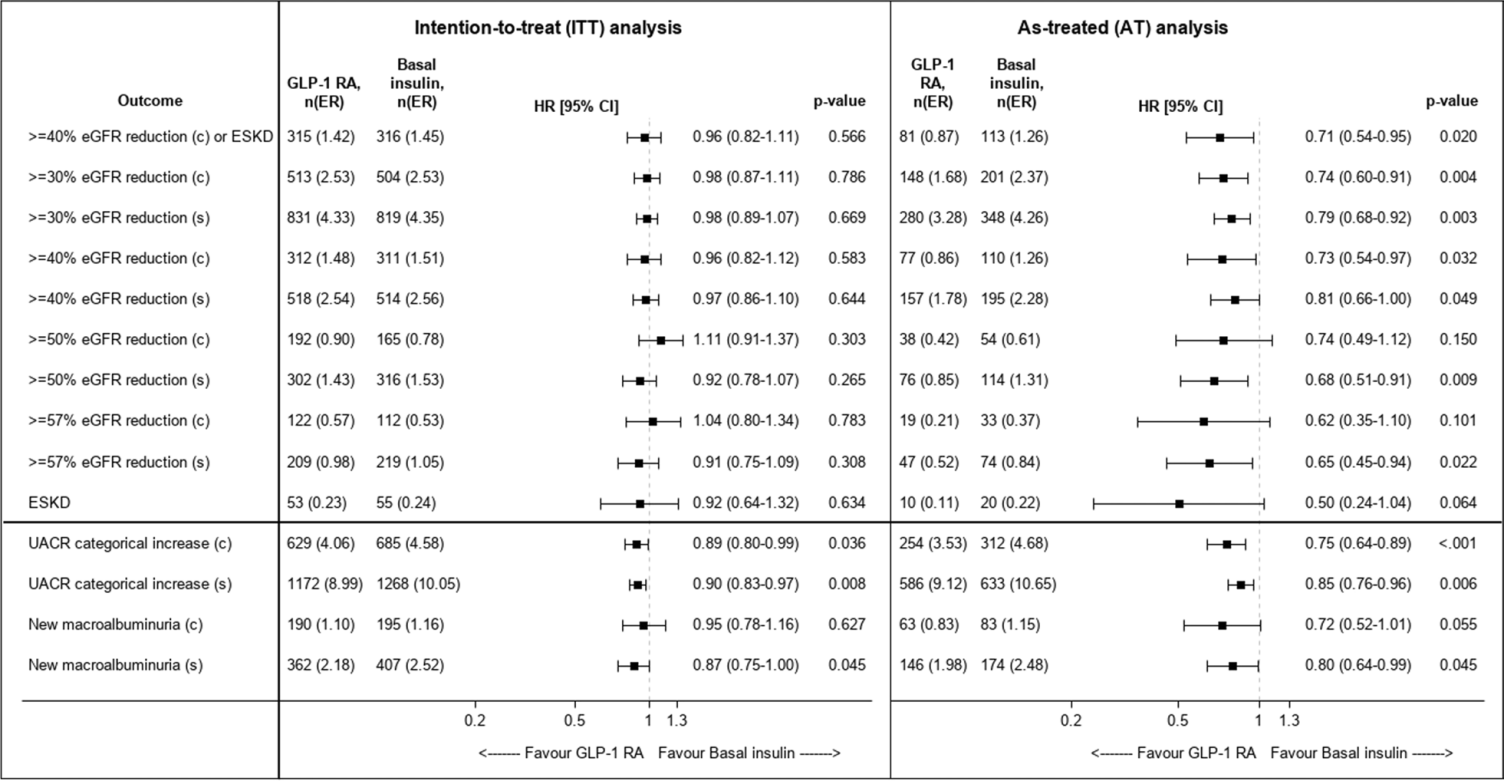
The association between initiation of GLP-1 RAs versus basal insulin and the risk of categorical eGFR decline or albuminuria progression in the ITT and AT analyses. The risks of GFR decline (by different thresholds) or albuminuria progression were compared between initiators of GLP-1 RAs and initiators of basal insulin. In the intention-to-treat (ITT) analysis, patients were followed until October 2021, end of data availability or death. In an as-treated (AT) analysis, follow-up was also censored at study-drug discontinuation or comparator-initiation. A categorical increase of UACR was defined for the following categories: < 30, 30- < 300 or ≥ 300 mg/g. New-onset macroalbuminuria was defined as UACR ≥ 300 among those with UACR ≤ 230 mg/g at baseline (resembling a ≥ 30% increase in UACR). Outcomes were assessed as single- or confirmed measurement. Cox proportional hazards regression models were applied to compare between treatment arms. Event rates are per 100 patients-years. *c* - confirmed measurement, *GLP-1 RAs* Glucagon-like peptide-1 receptor agonists, *eGFR* estimated glomerular filtration rate, *ER* event rate, *ESKD* End-stage kidney disease, *s* -single measurement, *UACR* Urine albumin-to-creatinine ratio

The risk of confirmed  ≥ 40% eGFR reduction was lower with GLP-1 RA compared with basal insulin only in the AT follow-up analysis (HR 0.73 [95% CI 0.54–0.97]), but not in the ITT (HR 0.96 [0.82–1.12]) or ITT-48mo (0.83 [0.63–1.10) analyses. Similar trends were observed for other categorical eGFR thresholds, whether defined as single- or confirmed-measurement (Fig. 3). The risk of ESKD was not different between the treatment groups (HR 0.92 [0.64–1.32] in the ITT analysis). All-cause mortality with GLP-1 RA versus basal insulin occurred at an incidence of 1.1 vs 1.5 and 0.5 vs 1.3 events per 100 patient’s years in the ITT and AT analyses, respectively. In the ITT analysis, GLP-1 RA’ initiation was associated with a lower risk for single- (HR 0.90 [0.83–0.97]) and confirmed—(0.89 [0.80–0.99]) categorical progression of UACR, compared with basal insulin (Fig. 3). The risk for single-measurement new onset macroalbuminuria was also lower with GLP-1 RA (0.87 [0.75–0.997]) but not the risk of confirmed new macroalbuminuria (0.95 [0.78–1.16]). Similar associations between initiation of GLP-1 RA versus basal insulin with albuminuria progression outcomes were observed for the ITT-48mo and AT analyses (Fig. 3 and Additional file 1: Figure S2).

### Change in eGFR over time

**Fig. 4:**
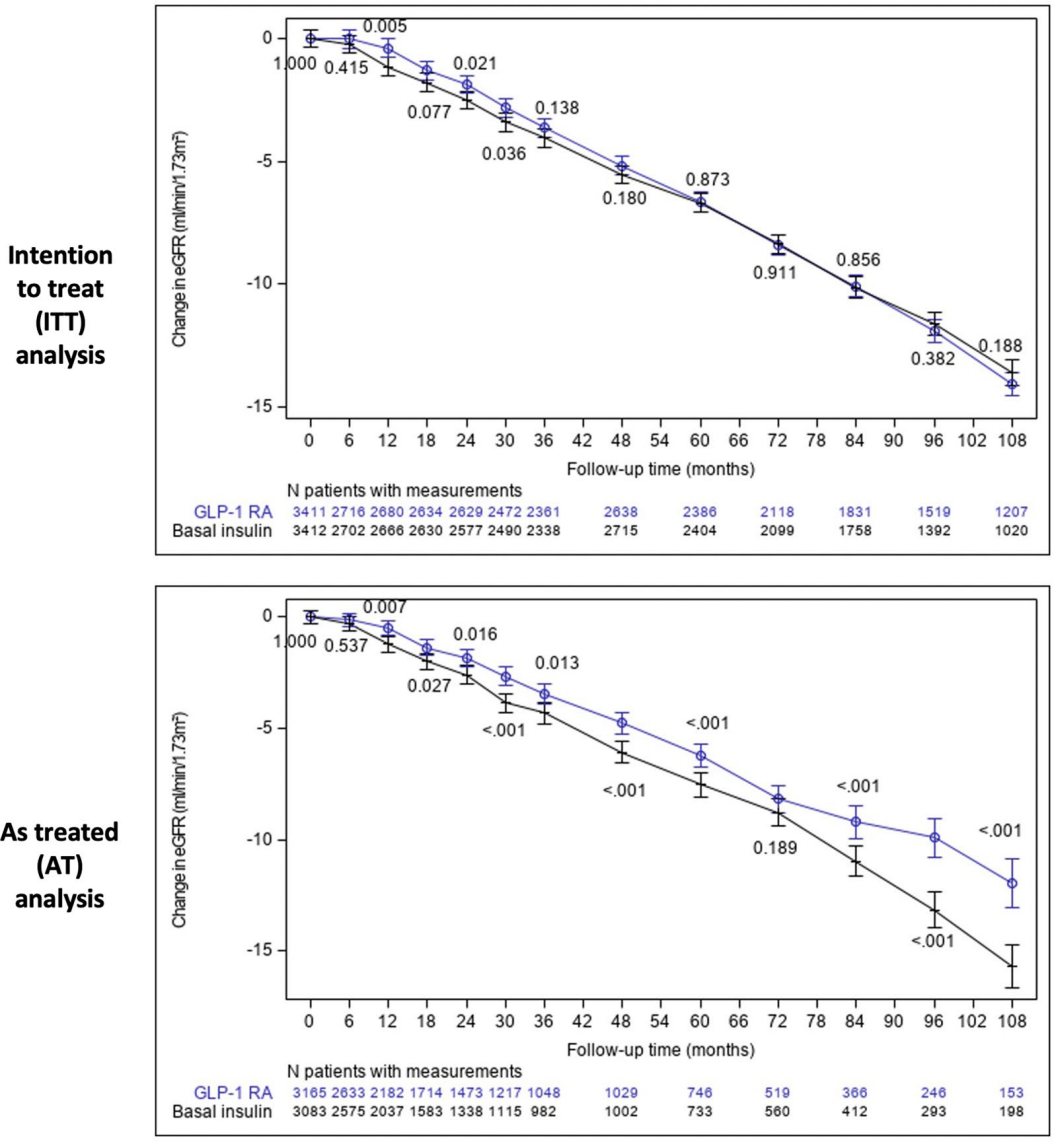
The change in eGFR overtime in initiators of GLP-1 RA versus basal insulin. At each time point (every 6 months for the first 3 years and then yearly), we considered the eGFR measurement closest to the end of the period for each patient. Mixed models with repeated measurements were applied to measure the change from baseline in eGFR at each time point and to calculate the p-value for the difference between groups (presented near each point). *GLP-1 RAs* Glucagon-like peptide-1 receptor agonists, *eGFR* estimated glomerular filtration rate

**Fig. 5:**
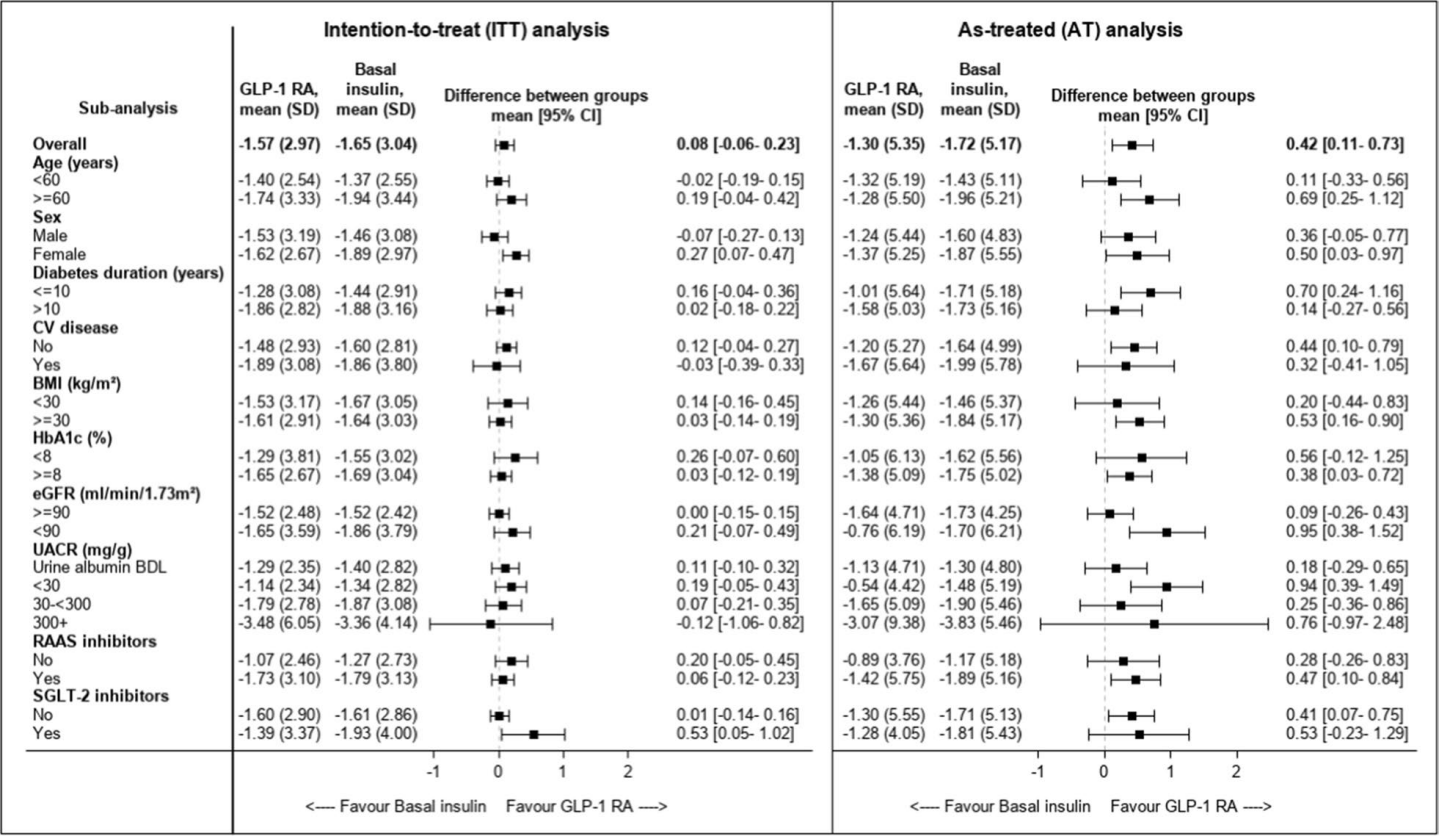
The change in eGFR overtime in initiators of GLP-1 RAs versus basal insulin in the ITT and AT analyses, by baseline subgroups. We calculated an eGFR slope per patient by fitting a linear regression model. We then calculated the mean eGFR slope over time for each group and used t-test to compare the treatment groups. eGFR slopes are presented as mL/min/1.73 m^2^/year. *BDL* below detectable levels, *BMI* body mass index, *CVD* cardiovascular disease, *eGFR* estimated glomerular filtration rate, *GLP-1 RAs* glucagon-like peptide 1 receptor agonist, *HbA1c* glycated hemoglobin A1c, *RAAS* renin angiotensin aldosterone system, *SGLT2i* sodium-glucose transporter 2 inhibitors, *UACR* urine albumin-to-creatinine ratio

Compared with basal insulin, initiators of GLP-1 RAs had mitigated eGFR loss in the AT analysis starting at 12 months and afterward at almost all time points (p ≤ 0.027). eGFR loss mitigation was observed in the ITT analysis only at time points 12, 24, and 30 months (p ≤ 0.036), but not in others (Fig. 4). Use of GLP-1 RAs was associated with a less steep eGFR slope compared with basal insulin in the AT analysis (mean annual between-group difference of 0.42 mL/min/1.73 m^2^/year [95% CI 0.11–0.73]; p = 0.008). No significant differences were observed in the whole study population in the ITT (0.08 mL/min/1.73 m^2^/year [−0.06 to 0.23]; p = 0.258) or ITT-48mo analyses (0.14 mL/min/1.73 m^2^/year [−0.03 to 0.30]; p = 0.103) (Figs. 4 and 5).

The between-group difference in the AT analysis was observed in patients with eGFR < 90 mL/min/1.73 m^2^ but not those with eGFR ≥ 90 mL/min/1.73 m^2^ (0.95 mL/min/1.73 m^2^/year [95% CI 0.38–1.52] and 0.09 mL/min/1.73 m^2^/year [-0.26–0.43], respectively). (Fig. 5 and Additional file 1: Table S6). In the AT analysis, the between group-differences in eGFR decline with GLP-1 RAs versus basal insulin was 0.53 [−0.23 to 1.28] and 0.41 mL/min/1.73 m^2^/year [0.07–0.74] in those with or without SGLT2i therapy at baseline (Fig. 5).

## Discussion

We used over a decade of real-world data to compare kidney outcomes between initiators of GLP-1 RAs and basal insulin. GLP-1 RAs initiators had a lower risk of albuminuria progression, compared with basal insulin. They also had a lower risk for a composite outcome of  ≥ 40% eGFR loss or ESKD, accompanied by mitigation of eGFR slope, in the AT, but not in the ITT analysis. The treatment effects seemed to be more pronounced in patients with eGFR  < 90 compared with  ≥ 90 mL/min/1.73 m^2^. All in all, these findings suggest that continuous use of GLP-1 RAs may be associated with lower kidney risk in a general population of patients with T2D, warranting further investigation.

Most data regarding the kidney effects of GLP-1 RAs come from cardiovascular outcome trials (CVOTs) enrolling patients with type 2 diabetes at high cardiovascular risk [12, 15] with a paucity of real-world data [18]. One study found a smaller eGFR decline after one year of treatment with GLP-1 RAs, compared to other GLAs [27]. In another study with a median follow-up duration of 1.0–1.5 years, the use of GLP-1 RAs was associated with a lower risk for the composite outcome of  > 50% eGFR decline, ESKD, or all-cause mortality, compared to either initiators of DPP4 inhibitors or sulfonylureas [28]. A longer study (mean follow-up of 3 years), found a lower risk for a composite outcome of kidney replacement therapy, or kidney failure-related hospitalization or death in initiators of GLP-1 RAs compared with DPP4i [29]. The current analysis adds several aspects to these studies. First, we use an injectable insulin comparator, which like GLP-1 RAs is often used in more advanced diabetes stages, in Israel and other countries. Second, owing to the large and granular number of eGFR samples during follow-up (median of 13 measurements over 84 months), we portrayed in detail the change in eGFR at different time points. Third, we assessed the annual eGFR slope over time with each treatment. Fourth, we confirm the favorable effects of GLP-1 RAs on albuminuria-based outcomes, as shown in clinical trials. Fifth, we provide important data regarding the kidney effects of GLP-1 RAs in patients already treated with SGLT2i.

In most CVOTs involving GLP-1 RA, the drugs reduced the risk of new macroalbuminuria compared to placebo [12, 15], however these trials sampled populations of patients at increased cardiovascular and kidney risk. It is unclear whether these benefits are transferable to the general population of patients with T2D with lower baseline cardiovascular and kidney risk, such as the participants in the current study. Due to the granular UACR data in MHS, we were able to analyze albuminuria outcomes. We found that compared with basal insulin, the use of GLP-1 RAs in real-world was associated with a lower risk for a single- and confirmed-categorical increase in UACR, or single-measurement new macroalbuminuria. The risk of confirmed new macroalbuminuria was not different between the groups. The lack of statistically significant effect on this outcome, which was repeatedly demonstrated to improve in the CVOTs with GLP-1 RAs [12], can be explained by the irregularity of UACR sampling in real-world setting compared with CVOTs. Of relevance, single-measurement albuminuria outcomes were shown to be sufficient to detect the treatment effects of drugs [30, 31]. Taken together, our findings suggest that the albuminuria-lowering effects of GLP-1 RAs observed in RCTs are also relevant in a general population of patients with type 2 diabetes with a lower cardiovascular and kidney risk profile.

When meta-analyzing data of CVOTs, the effects of GLP-1 RAs on worsening kidney function (excluding the albuminuria component) reached only marginal statistical significance with high heterogeneity among trials [12]. A recent pooled analysis of the LEADER and SUSTAIN-6 studies demonstrated a lower risk for persistent  ≥ 40% or ≥ 50% reduction in eGFR from baseline [32]. These effects were more pronounced in patients with eGFR < 60 mL/min/1.73 m^2^, or with UACR ≥ 30 mg/g. In the PIONEER 5 study enrolling patients with T2D and CKD, oral semaglutide use for one year versus placebo did not exert statistically significant changes in eGFR levels [33]. However, the AWARD-7 study demonstrated that in patients with T2D and CKD, dulaglutide mitigated eGFR loss compared with insulin glargine [34], especially in a subgroup of patients with macroalbuminuria at baseline [35]. Consistent with these data, we found that the association between GLP-1 RAs use and the kidney outcome seemed to be more pronounced in patients with lower eGFR. The ongoing FLOW trial investigates the kidney effects of once-weekly semaglutide s.c. in patients with T2D and CKD. Enrollment is stratified by treatment with SGLT2i at baseline (15.5% of the participants [17]). Findings of this study are expected to shed light on the effects of GLP-1 RA on kidney outcomes in patients with T2D and reduced kidney function, and whether SGLT2i use modifies these effects.

Studies from the past decade show that GLP-1 RA and SGLT2i are disease-modifying drugs with kidney and cardiovascular benefits that exceed their glucose-lowering effects [36]. Because these drugs were developed and studied simultaneously, there is limited long-term data on whether their combination has an additive cardiovascular or kidney protection. In our study, 950 patients received SGLT2i at baseline, more than in any CVOT with GLP-1RA [12]. The kidney benefits with GLP-1 RAs observed in the AT analysis did not seem to be mitigated in patients treated with SGLT2i at baseline, although the population was not stratified by the use of SGLT2i. In the AMPLITUDE-O trial, which was stratified by baseline SGLT2i therapy, the effects of efpeglenatide versus placebo on the cardiovascular and kidney outcomes were consistent by SGLT2i use [32]. Among the 618 (15.2%) participants who used SGLT2i at baseline, efpeglenatide improved the composite kidney outcome of incident macroalbuminuria, confirmed  ≥ 40% loss, or ESKD by 48% (95% CI [17–67]). These cumulating data are relevant to an important clinical question on whether to recommend the combined use of GLP-1 RA and SGLT2i for additional cardiovascular and kidney protection.

### Challenges and limitations

This is an observational study, and while we used different approaches to emulate an RCT setting, no causation can be concluded. Real-world analyses pose several challenges requiring careful expertise. In this study, we followed recent recommendations [37]. Namely, we used a predefined protocol with new-initiators design, defined specific outcomes, used an insulin comparator that is also indicated in advanced stages of type 2 diabetes, and applied PS matching to balance the cohorts. However, the baseline characteristics of the study cohorts before matching were unbalanced, and no statistical method is capable to completely emulate randomization, thus residual confounding cannot be excluded. Also, PS matching limits the generalizability of the results to populations that were dropped out in the process. We also predefined both ITT and AT analyses, each considers different aspects of real-world effectiveness of drugs. The ITT follow-up had a long median duration of 81.1 months. However, during most of the follow-up period, patients were not treated with the index drug alone—either stopping it or initiating the comparator drug, along with potential changes in other drugs during follow-up. Therefore, previous studies favored an AT analysis to assess treatment effects of eGFR slopes [38]. On the other hand, the AT analysis is prone to biases due to imbalanced censoring. For example, the event rate (per 100 patients-years) for mortality in the AT follow-up with basal insulin and GLP-1 RAs was 1.3 and 0.5, respectively. This difference is much more than shown in RCTs [12]. It may not be attributed directly to the treatment allocation, and is possibly explained by discontinuation of GLP-1 RA or initiation of insulin among patients who experience a pronounced deterioration in their clinical status. To address these challenges, we performed an additional analysis censoring the ITT follow-up at 4 years, emulating the LEADER study [13, 19]. This analysis still follows the ITT principle, but censoring is stopped when most participants are still on index treatment. The number of events in this analysis was relatively low owing to the low baseline risk of the population, reducing the power to detect treatment effects. However, the treatment effects were closer to those seen in the AT analysis. Finally, the dissimilarity between the baseline characteristics of the pre-matched cohorts, required extensive matching. Finaly, this study includes one healthcare organization, limiting the study's external validity to other populations or healthcare systems.

## Conclusion

Compared with basal insulin, initiation of GLP-1 RAs in a real-world setting is associated with a reduced risk of albuminuria progression and possible mitigation of kidney function loss in patients with T2D and mostly preserved kidney function. The association between GLP-1 RAs use and the kidney outcome seemed to be observed primarily among those with baseline eGFR < 90 mL/min/1.73 m^2^.

## Supplementary Information

**Additional file 1: Table S1**. Variable definitions, including ICD-9 and ATC codes, and MHS registries used for this study. **Table S2**. Baseline parameters in initiators of GLP-1 RAs or basal insulin before propensity-score matching. **Table S3**. Year of treatment initiation by study group, before and after matching. **Table S4**. Medianfollow-up duration per each follow-up analyses overall and by treatment arms. **Table S5.** Median number of eGFR measurements during follow-up per each follow-up analysis overall and by treatment arms. **Table S6**. Risk of the primary composite kidney outcomeor mean eGFR lossin patients with baseline eGFR > 90, 60- < 90, and < 60 mL/min/1.73 m^2^. **Figure S1.** CONSORT diagram describing the formation of the study population. **Figure S2**. The association between initiation of GLP-1 RA versus basal insulin and the risk of categorical eGFR decline or albuminuria progression in the ITT-48mo analyses.

## Data Availability

The datasets used and/or analysed during the current study are available from the corresponding author on reasonable request and pending study protocol and regulations.

## Abbreviations

ACEi: Angiotensin-converting enzyme inhibitors
ARB: Angiotensin receptor blocker
AT: As-treated
CKD: Chronic kidney disease
CKD-EPI: Chronic Kidney Disease Epidemiology Collaboration
CVD: Cardiovascular disease
CVOTs: Cardiovascular outcome trials
eGFR: Estimated glomerular filtration rate
ESKD: End-stage kidney disease
GLAs: Glucose-lowering agents
GLP-1 RAs: Glucagon-like peptide-1 receptor agonists
HMO: Healthcare maintenance organization
HR: Hazard ratio
IRB: Institutional review board
IQR: Interquartile range
ITT: Intention-to-treat
MHS: Maccabi Healthcare Services
MRA: Mineralocorticoid receptor antagonist
PS: Propensity score
RCTs: Randomized controlled trials
SES: Socioeconomic status
STD: Standardized difference
T2D: Type 2 diabetes
UACR: Urine-albumin-to-creatinine ratio
