# Supplementary material for: Kidney function loss and albuminuria progression with GLP-1 receptor agonists versus basal insulin in patients with type 2 diabetes: real-world evidence
Source: Cardiovasc Diabetol. 2023 May 27;22:126. doi: 10.1186/s12933-023-01829-0 (PMC10225085; doi:10.1186/s12933-023-01829-0)
Supplement: Supplementary file 1 — Additional file 1: Table S1. Variable definitions, including ICD-9 and ATC codes, and MHS registries used for this study. Table S2. Baseline parameters in initiators of GLP-1 RAs or basal insulin before propensity-score matching. Table S3. Year of treatment initiation by study group, before and after matching. Table S4. Medianfollow-up duration per each follow-up analyses overall and by treatment arms. Table S5. Median number of eGFR measurements during follow-up per each follow-up analysis overall and by treatment arms. Table S6. Risk of the primary composite kidney outcomeor mean eGFR lossin patients with baseline eGFR > 90, 60- < 90, and < 60 mL/min/1.73 m2. Figure S1. CONSORT diagram describing the formation of the study population. Figure S2. The association between initiation of GLP-1 RA versus basal insulin and the risk of categorical eGFR decline or albuminuria progression in the ITT-48mo analyses. [file 12933_2023_1829_MOESM1_ESM.docx]

**Kidney function loss and albuminuria progression with GLP-1 receptor agonists versus basal insulin in patients with type 2 diabetes: real-world evidence**

Meir Schechter* ^1,2,3^, Cheli Melzer Cohen* ^4^, Alisa Fishkin ^1,2^, Aliza Rozenberg ^1,2^, Ilan Yanuv ^1,2^, Dvora R. Sehtman-Shachar^1,2^, Gabriel Chodick ^4,5^, Alice Clark ^6^, Trine J. Abrahamsen ^6^, Jack Lawson ^6^, Avraham Karasik ^4,7^, Ofri Mosenzon ^1,2^

* These authors have equally contributed

^1^ Diabetes Unit, Department of Endocrinology and Metabolism, Hadassah Medical Center, Jerusalem, Israel

^2^ Faculty of Medicine, Hebrew University of Jerusalem, Jerusalem, Israel

^3^ Department of Clinical Pharmacy and Pharmacology, University Medical Center Groningen, University of Groningen, Groningen, The Netherlands

^4^ Maccabi Institute for Research and Innovation, Maccabi Healthcare Services, Tel-Aviv, Israel

^5^ School of Public Health Sackler, Faculty of Medicine, Tel Aviv University, Tel Aviv, Israel

^6^ Novo Nordisk A/S, Copenhagen, Denmark

^7^ Tel Aviv University, Tel Aviv, Israel

Corresponding author:

Ofri Mosenzon MD MSc

OFRIM@hadassah.org.il

The Diabetes Unit, Department of Endocrinology and Metabolism

Hadassah Ein Kerem Medical Center

P.O.B 12000

Jerusalem 9112001

Israel

Supplementary methods:

For propensity score matching we used the following 88 baseline variables:

**Demographics:** Age, sex, socioeconomic status (1-3, 4-5, 6-7, 8-10), time of entering into study (by year and quartiles), body mass index (<25, 25-<30, 30-<35, 35-<40, ≥40, missing; kg/m^2^), smoking status (current smoker, past smoker, never smoker, missing), systolic blood pressure, diastolic blood pressure.

**Co-morbidities**: duration (years) with diabetes (≤2, 2-5, 5-10, ≥10), ischemic heart disease, myocardial infarction or after cardiac revascularization procedure, unstable angina, stable angina, atrial fibrillation, cerebrovascular disease, stroke, transient ischemic attack, peripheral vascular disease, bariatric surgery, hypertension, hyperlipidemia, cancer, diabetic neuropathy, diabetic nephropathy, diabetic retinopathy or other ophthalmic manifestations, diabetic foot or lower extremity amputation, liver disease, osteoarthritis, obstructive sleep apnea, chronic obstructive pulmonary disease (COPD), hypothyroidism, anxiety, depression, psychoses.

**Medications:** metformin, sulfonylureas, thiazolidinediones, meglitinides, alpha-glucosidase inhibitors, sodium-glucose cotransporter 2 inhibitors, fast-acting insulin, angiotensin converting enzyme (ACE) inhibitor, Angiotensin II receptor blocker, antihypertensive drugs, calcium channel blocker, thiazides, loop diuretics, other diuretics, nitrates, other hypertension drugs, statins, PCSK-9 inhibitors, other lipid-lowering drugs (excluding statins), COPD or asthma medications, antiplatelet, anticoagulants, heparin and low-molecular weight heparins, oral corticosteroids, nonsteroidal anti-inflammatory drug, opioids, antidepressants, antipsychotics, anti-parkinson’s agents, mineralocorticoid receptor antagonists, digoxin, anti-arrhythmic drugs, bisphosphonates, anti-convulsants, benzodiazepines, proton pump inhibitors.

**Laboratory examinations:** estimated glomerular filtration rate (eGFR), urine-albumin-to-creatinine ratio (UACR; below detectable, <15, 15-<30, 30-300, ≥300, missing), total cholesterol, low-density cholesterol, high-density cholesterol, triglycerides, baseline eGFR slope, Fasting-plasma glucose, Alanine transaminase (ALT), aspartate transaminase (AST), alkaline phosphatase, blood urea nitrogen, platelets count, haemoglobin, serum albumin, sodium, potassium, calcium.

**Table S1**: Variable definitions, including ICD-9 and ATC codes, and MHS registries used for this study.

| **Covariate** | **Definition (when applicable)** | **Codes** |
| --- | --- | --- |
| Age | Age at treatment initiation |  |
| Sex | female, male |  |
| Weight | Last evaluation during baseline period |  |
| Height | Last evaluation during baseline period |  |
| Socioeconomic status | By Category SES 1-10 at baseline period |  |
| Blood pressure | Last evaluation during baseline period at baseline period |  |
| Smoking status | Last recorded status during baseline period |  |
| **Co-morbidities** |  |  |
| ASCVD disease | Entered into CVD major registry anytime prior to index date |  |
| Ischemic heart disease | Entered into Ischemic heart disease registry anytime prior to index date |  |
| Myocardial infarction registry | Entered into MI registry anytime prior to index date |  |
| Myocardial infarction | ICD-9 CM anytime prior to index date. | ICD-9 codes 410.x |
| PCI (Diagnostic and therapeutic) | ICD-9 CM and CPT codes anytime prior to index date. | ICD-9 codes: 00.66, V45.82, 36.0  CPT codes: 92980, 92982, 92984 |
| CABG | ICD-9 CM and CPT codes anytime prior to index date. | ICD-9 codes: V45.81, 36.1  CPT codes: 33510, 33999 (with specific character codes) |
| Cerebrovascular disease | Entered into Cerebrovascular disease registry prior to index date |  |
| Stroke | Entered into Stroke registry prior to index date. |  |
| Transient ischemic attack | Entered into TIA registry prior to index date |  |
| Peripheral vascular disease | Entered into PVD registry prior to index date or with recorded procedure base on CPT or ICD-9 CM codes | ICD-9 codes: 440.20 – 440.24, 440.29 – 440.32, 440.3, 440.4, 443.9, 38.18, 38.19 , '39.25', '39.29' , '38.08', '38.09', '38.38', '38.39','38.48', '38.49', '39.5X' , '39.9' ; CPT codes: 35200, 35220 , 75962 , 78445, 3556 |
| Congestive heart disease | Entered into CHF registry prior to 1.11.2021 | ICD-9 codes |
| Atrial fibrillation | Entered into Atrial fibrillation registry prior to 1.11.2021 |  |
| unstable angina | Anytime prior index date. | ICD-9 code: 411.x |
| Stable angina | Anytime prior index date. | ICD-9 code: 413.x |
| Bariatric surgery | Anytime prior index date. | CPT codes: 43644, 43842, 43845, 43848 |
| Hypertension | Entered into Hypertension registry anytime prior to index date |  |
| Hyperlipidemia | At baseline period. | 272.0x-272.4x |
| Cancer | Entered into cancer registry anytime prior to index date |  |
|  | At baseline period | ICD-9 codes: 607.84 |
| Diabetes mellitus | Entered into diabetes registry anytime prior to 1.1.2020 | Diabetes type was based on registrie's classification. Among patients without classification, if they were on fast acting insulin, ther they considered as type 1. Otherwise, as type 2. |
| Diabetic neuropathy | Anytime prior index date | ICD-9 codes: 250.6x, 357.2x, 337.1 |
| Diabetic nephropathy | Anytime prior index date | ICD-9 codes: 250.4x, 583.81 |
| Diabetic foot or lower extremity amputation | Anytime prior index date | ICD-9 codes: 707.1x, V49.7x (excluding V49.76 or V49.77)  Procedures: 84.10-84.17  CPT codes: 27590, 27880, 27884, 28800, 28820 |
| Diabetic retinopathy or other ophthalmic manifestations | Anytime prior index date | ICD-9 codes: 362.0x, 250.5x (without 362.01-362.07), 366.41 (diabetic cataract), 365.44 (diabetic glaucoma), 361.9x, 379.23,  Or procedures: 14.7x, 14.24, 14.34, 14.54 |
| Liver disease | Anytime prior index date | ICD-9 codes: 070.x, 570.x- 573.x 456.0x-456.2x, 576.8x, 782.4x, 789.5x, 39.1x, 42.91 |
| End stage kidney disease or on dialysis | Based in eGFR evaluations, CKD registry, procedures or based on ICD-9 CM codes (Anytime [except of eGFR evaluations]) | Defined on stage 5, on dialysis in the past or after kidney transplantation; eGFR<15; ICD-9 CM codes: 585.5x, 585.6x , V56.0x, V56.8x, V45.1x , 39.95, 54.98 , V42.0x, 996.81 , 55.6x or on dialysis |
| Dialysis | Anytime prior index date | ICD-9 CM code: 39.95 , V45.1 , V56.0 , V56.1 ; CPT codes: 90935, 90945 or as defined in CKD registry |
| Acute kidney injury that requires dialysis (ARF-D) | Anytime prior index date | ICD-9 CM code: 39.95 , V45.1 , V56.0 , V56.1 ; CPT codes: 90935, 90945 |
| Osteoarthritis | At baseline period | ICD-9 codes: 715.x |
| Obstructive Sleep Apnea | At baseline period | ICD-9 codes: 327.23 |
| Hypothyroidism | At baseline period | ICD-9 codes: 244.X |
| COPD | Entered into COPD registry anytime prior to index date |  |
| Anxiety | At baseline period | ICD-9 codes: 293.84, 300.0x, 300.2x, 300.3x, 309.24, 308.0x, 309.81 |
| Depression | At baseline period | ICD-9 codes: 293.83, 296.2x. 296.3x, 298.0x, 300.4x, 309.0x, 309.1x, 309.28, 311.xx |
| Psychoses | At baseline period | ICD-9 codes: 290.8x, 290.9x, 295.xx, 297.xx, 298.xx, 299.xx, 780.1x |
| Gestational ultrasound | 9 months before index date and during treatment | CPT codes: 58974, 59000, 76801, 76805, 76810, 76811, 76815, 76816, 76817, 78657 |
| **Concomitant medications** |  |  |
| Angiotensin-converting-enzyme inhibitor | ≥1 filled dispensation/any other record of the drug use at baseline period | ATC codes: C09A Angiotensin-converting-enzyme inhibitors, plain C09B Angiotensin-converting-enzyme inhibitors, combinations |
| Angiotensin II receptor blocker | ≥1 filled dispensation/any other record of the drug use at baseline period | ATC codes: C09C angiotensin ii antagonists, plain C09D angiotensin ii antagonists, combinations |
| Beta-blocker | ≥1 filled dispensation/any other record of the drug use at baseline period | ATC codes: C07 beta-blocking agents |
| Calcium channel blocker | ≥1 filled dispensation/any other record of the drug use at baseline period | ATC codes: C08 calcium channel blockers C07FB Beta-blocking agents and calcium channel blockers C09BB Angiotensin-converting-enzyme inhibitors and calcium channel blockers C09DB Angiotensin II antagonists and calcium channel blockers |
| Thiazides | ≥1 filled dispensation/any other record of the drug use at baseline period | ATC codes: C03A low-ceiling diuretics, thiazides |
| Loop diuretics | ≥1 filled dispensation/any other record of the drug use at baseline period | ATC codes: C03C high-ceiling diuretics |
| Other diuretics | ≥1 filled dispensation/any other record of the drug use at baseline period | ATC codes: C03D potassium-sparing agents |
| Nitrates | ≥1 filled dispensation/any other record of the drug use at baseline period | ATC codes: C01DA02 glyceryl trinitrate C01DA08 isosorbide dinitrate C01DA14 isosorbide mononitrate C01EB18 ranolazine |
| Other hypertension drugs | ≥1 filled dispensation/any other record of the drug use at baseline period | ATC codes: C02CA04 doxazosin C03DA04 eplerenone C02CA01 prazosin G04CA03 terazosin C02AC01 clonidine C02AC02 guanfacine C02DB02 hydralazine C09XA02 aliskiren C09XA52 aliskiren and hydrochlorothiazide |
| PCSK-9 inhibitors | ≥1 filled dispensation/any other record of the drug use at baseline period | ATC codes: C10AX13 Evolocumab Bococizumab C10AX14 Alirocumab |
| Statins | ≥1 filled dispensation/any other record of the drug use at baseline period | ATC codes: C10AA |
| Other lipid-lowering drugs, excluding statins | ≥1 filled dispensation/any other record of the drug use at baseline period | ATC codes: C10 lipid modifying agents, excluding  C10AA, C10BA, C10BX |
| COPD or asthma medications | ≥1 filled dispensation/any other record of the drug use at baseline period | ATC codes: R03AK06,R03AK07, R03AC13, R03AC12, R03AC02, R03CC03, R01AX03, R03BB01, R03BB04, R03DA04, R03DC03, R03DC01, R03BB05, R03AL05, R03AC18, R03AC19, R03BB07, R03AL01, R03AL02, R03AL03, R03AL04, R03AL05, R03AL06, R03AL08, R03AL09 |
| Antiplatelet | ≥1 filled dispensation/any other record of the drug use at baseline period | ATC codes: B01AC06 , B01AC04 , B01AC22 , B01AC05 , B01AC07 , B01AC23 , B01AC24 |
| Anticoagulants | ≥1 filled dispensation/any other record of the drug use at baseline period | ATC codes: B01AA03 , B01AE07 , B01AF01 , B01AF02 |
| Heparin and other low-molecular weight heparins | ≥1 filled dispensation/any other record of the drug use at baseline period | ATC codes: B01AB , B01AE07 |
| Oral corticosteroids | ≥1 filled dispensation/any other record of the drug use at baseline period | ATC codes: H02AB10 , H02AB09, H02AB07, H02AB06, H02AB04, H02AB08, H02AB02, H02AB01 |
| NSAIDs | ≥1 filled dispensation/any other record of the drug use (only prescription medications) at baseline period | ATC codes: M01A requiring |
| Metformin | ≥1 filled dispensation/any other record of the drug use at baseline period | ATC codes: A10BA02, A10BD07, A10BD08, A10BD10, A10BD11, A10BD13, |
| Sulfonylureas 2nd generation | ≥1 filled dispensation/any other record of the drug use at baseline period | ATC codes: A10BB |
| Dipeptidyl peptidase-4 inhibitors | ≥1 filled dispensation/any other record of the drug use at baseline period | ATC codes: A10BH, A10BD07, A10BD08, A10BD10, A10BD11, A10BD13, A10BD19, A10BD21, A10BD25 |
| Glucagon-like peptide-1 receptor agonists | ≥1 filled dispensation/any other record of the drug use at baseline period | ATC codes: A10BJ |
| Thiazolidinediones | ≥1 filled dispensation/any other record of the drug use at baseline period | ATC codes: A10BG |
| Meglitinides | ≥1 filled dispensation/any other record of the drug use at baseline period | ATC codes: A10BX02,  A10BX03 |
| Insulin | ≥1 filled dispensation/any other record of the drug use at baseline period | ATC codes: A10A insulins and analogs |
| Alpha-glucosidase inhibitors | ≥1 filled dispensation/any other record of the drug use at baseline period | ATC codes: A10BF |
| SGLT-2i | ≥1 filled dispensation/any other record of the drug use at baseline period | ATC codes: A10BD15, A10BD19, A10BD20, A10BD21, A10BD25 |
| Opioids | ≥1 filled dispensation/any other record of the drug use at baseline period | ATC codes: N02A |
| Antidepressants | ≥1 filled dispensation/any other record of the drug use at baseline period | ATC codes: N06A |
| Antipsychotics | ≥1 filled dispensation/any other record of the drug use at baseline period | ATC codes: N05A |
| Agents for dementia | ≥1 filled dispensation/any other record of the drug use at baseline period | ATC codes: N06D |
| Antiparkinson agents | ≥1 filled dispensation/any other record of the drug use at baseline period | ATC codes: N04 |
| Mineralocorticoid receptor antagonists (MRAs; aldosterone antagonists) | ≥1 filled dispensation/any other record of the drug use at baseline period | ATC codes: C03Da |
| Digoxin | ≥1 filled dispensation/any other record of the drug use at baseline period | ATC codes: C01AA05 |
| Neprilysin inhibitors (Valsartan and sacubitril) | ≥1 filled dispensation/any other record of the drug use at baseline period | ATC codes: C09DX04 |
| Anti- arrhythmic drugs | ≥1 filled dispensation/any other record of the drug use at baseline period | ATC codes: C01B |
| Lithium | ≥1 filled dispensation/any other record of the drug use at baseline period | ATC codes: N05AN |
| Heparin/LMWH | ≥1 filled dispensation/any other record of the drug use at baseline period | ATC codes: B01AB |
| Bisphosphanates | ≥1 filled dispensation/any other record of the drug use at baseline period | ATC codes: M05BA |
| Anti-convulsant | ≥1 filled dispensation/any other record of the drug use at baseline period | ATC codes: N03 |
| Benzodiazepines | ≥1 filled dispensation/any other record of the drug use at baseline period | ATC codes: N03AE, N05B, N05C |
| Proton pump inhibitors | ≥1 filled dispensation/any other record of the drug use at baseline period | ATC codes: A02BC |
| **Laboratory examinations** |  |  |
| Fasting plasma glucose (mg/dl) | Last measurement during baseline period |  |
| Hemoglobin | Last measurement during baseline period |  |
| eGFR slope | slope of all eGFR evaluation during a baseline period of maximum 4 years, when there is at-least 1 measurement during the 12-months baseline period, and there are at least 180 days between the first and last evaluation (not necessary consecutive) |  |
| Plasma electrolytes | Plasma potassium, sodium, chloride, magnesium and potassium. Last measurement during baseline period |  |
| Plasma albumin | Last measurement during baseline period |  |
| HbA1c (%) | Last measurement during baseline period |  |
| Total cholesterol (mg/dl) | Last measurement during baseline period |  |
| High-density lipoprotein (HDL) level (mg/dl) | Last measurement during baseline period |  |
| Low-density lipoprotein (LDL) level (mg/dl) | Last measurement during baseline period |  |
| Triglyceride level (mg/dl) | Last measurement during baseline period |  |
| Creatinine (mg/dl) | eGFR was calculated from serum creatinine by using 2009 CKD-epi equation. |  |
| UACR (mg/g) | All evaluation since 4 years before index date |  |
| Blood urea (mg/dl) | Last measurement during baseline period |  |
| Liver Function test | Laboratory tests of: ALT, AST, Alkaline Phosphatase, bilirubin total. Last measurement during baseline period |  |
| Platelets count | Last measurement during baseline period |  |

Abbreviations: ALT- alanine transaminase; ARF-D- Acute renal failure (that requires) dialysis; ASCVD – atherosclerotic cardiovascular disease; AST- aspartate aminotransferase; ATC code-  anatomical therapeutic chemical code; CABG- coronary artery bypass graft ; CHF – congestive heart failure; CKD – chronic kidney disease; COPD- chronic obstructive pulmonary disease; CPT – current procedural terminology; CVD – cardiovascular disease ; eGFR – estimated glomerular filtration rate; HbA1c – hemoglobin A1c; ICD9-CM - international classification of diseases, ninth revision, clinical modification; LMWH – low molecular weight heparin ;MI – myocardial infraction; NSAIDs- non-steroidal anti-inflammatory drugs; PCSK9- Proprotein convertase subtilisin/kexin type 9 ; PVD – peripheral vascular disease; SES – socioeconomic status; SGLT-2i- sodium-glucose transporter 2 inhibitor; TIA – transient ischemic attack; UACR- urinary albumin to creatinine ratio.

**Table S2**: Baseline parameters in initiators of GLP-1 RA or basal insulin before propensity-score matching

| **characteristic** | **Level** | **GLP-1 RA (n=6797)** | **Basal insulin (n=9145)** | **STD** |
| --- | --- | --- | --- | --- |
| **Demographic characteristics** |  |  |  |  |
| **Age (years)** | Mean (SD) | 59.2 (9.5) | 59.3 (10.8) | -0.02 |
| **Women (%)** | n (%) | 3118 (45.9) | 3656 (40.0) | 0.12 |
| **Socioeconomic status** | 1-3, n (%) | 822 (12.1) | 1442 (15.8) | 0.18 |
|  | 4-5, n (%) | 1912 (28.1) | 2907 (31.8) |  |
|  | 6-7, n (%) | 2387 (35.1) | 3068 (33.5) |  |
|  | 8-10, n (%) | 1670 (24.6) | 1711 (18.7) |  |
|  | Missing, n (%) | 6 (0.1%) | 17 (0.2%) |  |
|  | Mean (SD) | 6.0 (2.0) | 5.7 (1.9) | 0.18 |
| **Medical history** |  |  |  |  |
| **Years in diabetes registry** | Mean (SD) | 9.7 (4.6) | 9.4 (4.9) | 0.07 |
| **Established CVD history*** | n (%) | 1327 (19.5) | 1947 (21.3) | -0.04 |
| **Hypertension registry*** | n (%) | 4851 (71.4) | 5701 (62.3) | 0.19 |
| **BMI kg/m²** | Mean (SD) | 34.6 (5.2) | 30.3 (5.7) | 0.78 |
|  | Missing, n (%) | 176 (2.6) | 572 (6.3) |  |
| **HbA1c (%)** | Mean (SD) | 8.5 (1.3) | 9.7 (1.9) | -0.72 |
|  | Missing, n (%) | 5 (0.1) | 42 (0.5) |  |
| **Medications** |  |  |  |  |
| **Metformin** | n (%) | 6617 (97.4) | 8784 (96.1) | 0.07 |
| **Sulfonylureas 2nd generation** | n (%) | 3181 (46.8) | 5104 (55.8) | -0.18 |
| **SGLT2i** | n (%) | 1791 (26.3) | 819 (9.0) | 0.47 |
| **RAAS inhibitors** | n (%) | 5165 (76.0) | 6326 (69.2) | 0.15 |
| **Thiazolidinediones** | n (%) | 524 (7.7) | 560 (6.1) | 0.06 |
| **Fast acting insulin** | n (%) | 38 (0.6) | 558 (6.1) | -0.31 |
| **Beta blockers** | n (%) | 2623 (38.6) | 3116 (34.1) | 0.09 |
| **Aldosterone antagonists** | n (%) | 198 (2.9) | 230 (2.5) | 0.02 |
| **Antihypertensives** | n (%) | 5484 (80.7) | 6792 (74.3) | 0.15 |
| **Kidney markers** |  |  |  |  |
| **eGFR (ml/min/1.73m²)** | >90, n (%) | 4241 (62.4) | 5520 (60.4) | 0.23 |
|  | 60-90, n (%) | 2081 (30.6) | 2526 (27.6) |  |
|  | 45-60, n (%) | 369 (5.4) | 588 (6.4) |  |
|  | 30-45, n (%) | 95 (1.4) | 426 (4.7) |  |
|  | 15-30, n (%) | 11 (0.2) | 85 (0.9) |  |
|  | Mean (SD) | 91.3 (18.0) | 89.6 (22.0) | 0.09 |
| **UACR (mg/g)** | Below detectable, n (%) | 2502 (36.8) | 3064 (33.5) | 0.15 |
|  | <15, n (%) | 878 (12.9) | 1063 (11.6) |  |
|  | 15-<30, n (%) | 923 (13.6) | 1139 (12.5) |  |
|  | 30-300, n (%) | 1705 (25.1) | 2372 (25.9) |  |
|  | >=300, n (%) | 447 (6.6) | 789 (8.6) |  |
|  | Missing, n (%) | 342 (5.0) | 718 (7.9) |  |
|  | Median (IQR) | 13.3 (0.0- 49.2) | 15.9 (0.0- 62.3) | -0.09 |

*Based on MHS registries.

Abbreviations: BMI **–** body mass index; CVD **–** cardiovascular disease; eGFR **–** estimated glomerular filtration rate; GLP-1 RA – glucagon-like peptide 1 receptor agonist; RAAS – renin angiotensin aldosterone system; SGLT2i – sodium-glucose transporter 2 inhibitor; STD, standardized difference; UACR **–** urinary albumin to creatinine ratio; MHS – Maccabi Healthcare Services.

**Table S3**: Year of treatment initiation by study group, before and after matching

|  | **Before propensity-score matching** | | | **After propensity-score matching** | | |
| --- | --- | --- | --- | --- | --- | --- |
| **Year of treatment initiation** | **GLP-1 RA (n=6797)** | **Basal insulin (n=9145)** | **STD** | **GLP1-RA (n=3424)** | **Basal insulin (n=3424)** | **STD** |
| 2010, n (%) | 394 (5.8) | 994 (10.9) | 0.50 | 320 (9.3) | 346 (10.1) | 0.00 |
| 2011, n (%) | 559 (8.2) | 1057 (11.6) |  | 416 (12.1) | 328 (9.6) |  |
| 2012, n (%) | 687 (10.1) | 1120 (12.2) |  | 483 (14.1) | 353 (10.3) |  |
| 2013, n (%) | 518 (7.6) | 1121 (12.3) |  | 325 (9.5) | 407 (11.9) |  |
| 2014, n (%) | 560 (8.2) | 1105 (12.1) |  | 345 (10.1) | 377 (11.0) |  |
| 2015, n (%) | 536 (7.9) | 1089 (11.9) |  | 303 (8.8) | 388 (11.3) |  |
| 2016, n (%) | 579 (8.5) | 817 (8.9) |  | 266 (7.8) | 351 (10.3) |  |
| 2017, n (%) | 783 (11.5) | 700 (7.7) |  | 281 (8.2) | 321 (9.4) |  |
| 2018, n (%) | 864 (12.7) | 581 (6.4) |  | 280 (8.2) | 287 (8.4) |  |
| 2019, n (%) | 1317 (19.4) | 561 (6.1) |  | 405 (11.8) | 266 (7.8) |  |

Abbreviation: STD, standardized difference.

**Table S4**: Median (IQR) follow-up duration per each follow-up analyses overall and by treatment arms

|  |  | **Intention to treat analysis** | | **As-treated analysis** | | **ITT-48 months (4 years)** | |
| --- | --- | --- | --- | --- | --- | --- | --- |
| **Group** | **N** | **Median [IQR]** | **PY** | **Median [IQR]** | **PY** | **Median [IQR]** | **PY** |
| **GLP-1 RAs** | 3424 | 83.4 [50.0-112.4] | 23123.3 | 23.9 [9.2-47.7] | 9443.0 | 48.0 [48.0-48.0] | 12678.5 |
| **Basal insulin** | 3424 | 79.7 [51.6-106.5] | 22654.6 | 19.8 [6.9-46.1] | 9164.3 | 48.0 [48.0-48.0] | 12762.6 |
| **Total** | 6848 | 81.1 [50.9-110.0] | 45777.8 | 22.3 [8.4-46.8] | 18607.2 | 48.0 [48.0-48.0] | 25441.1 |

Legends: In an intention-to-treat (ITT) analysis, patients were followed until October 2021, end of data availability or death. In an ITT-48mo analysis, to emulate the LEADER study, we censored follow-up also at 4 years. In an as-treated (AT) analysis, follow-up was also censored at study-drug discontinuation or comparator-initiation.

Abbreviations: GLP-1 RAs – Glucagon-like peptide-1 receptor agonists; PY – Patients years.

**Table S5:** Median number of eGFR measurements during follow-up per each follow-up analysis overall and by treatment arms

|  | **GLP-1 RA** | **Basal insulin** | **Entire population** |
| --- | --- | --- | --- |
| **Follow-up duration** | **Median [IQR]** | **Median [IQR]** | **Median [IQR]** |
| **Intention-to-treat** | 14 [7-22] | 13 [7-20] | 13 [7-21] |
| **As-treated** | 4[2-8] | 4 [1-8] | 4 [1-8] |
| **intention-to-treat- 48 months** | 8 [5-11] | 8 [5-11] | 8 [5-11] |

Abbreviations: GLP-1 RAs – Glucagon-like peptide-1 receptor agonists.

**Table S6**: Risk of the primary composite kidney outcome (≥40% eGFR loss or ESKD) (**A**) or mean eGFR loss (**B**) in patients with baseline eGFR >90, 60-<90, and <60 mL/min/1.73 m^2^

A. Risk of the primary composite kidney outcome

|  | **Intention to treat analysis** | | | | | **As-treated analysis** | | | | |
| --- | --- | --- | --- | --- | --- | --- | --- | --- | --- | --- |
| **category** | **GLP-1 RA, n (ER)** | **Basal Insulin, n (ER)** | **HR [95%CI]** | **p-value** | **p-interaction** | **GLP-1 RA, n (ER)** | **Basal Insulin, n (ER)** | **HR [95%CI]** | **p-value** | **p-interaction** |
| ≥90 | 122 (0.9) | 97 (0.7) | 1.21 [0.93-1.58] | 0.160 | 0.083 | 30 (0.5) | 24 (0.5) | 1.2 [0.70-2.05] | 0.502 | 0.129 |
| 60-90 | 135 (2.0) | 152 (2.4) | 0.81 [0.64-1.03] | 0.080 |  | 35 (1.2) | 62 (2.1) | 0.59 [0.39-0.89] | 0.012 |  |
| <60 | 58 (3.8) | 67 (4.1) | 0.94 [0.66-1.34] | 0.730 |  | 16 (2.2) | 27 (3.2) | 0.79 [0.42-1.48] | 0.462 |  |

B. Mean annual eGFR loss

|  | **Intention to treat analysis** | | | | **As-treated analysis** | | | |
| --- | --- | --- | --- | --- | --- | --- | --- | --- |
| **category** | **GLP-1 RA slope, mean (SD)** | **Basal insulin slope, mean (SD)** | **Mean difference [95% CI]** | **p-value** | **GLP-1 RA slope, mean (SD)** | **Basal insulin slope, mean (SD)** | **Mean difference [95% CI]** | **p-value** |
| >=90 | -1.52 (2.48) | -1.52 (2.42) | 0 [-0.15-0.15] | 0.965 | -1.64 (4.71) | -1.73 (4.25) | 0.09 [-0.26-0.43] | 0.622 |
| 60-90 | -1.76 (3.14) | -1.98 (3.98) | 0.22 [-0.09-0.53] | 0.167 | -0.87 (5.88) | -1.8 (6.51) | 0.93 [0.28-1.59] | 0.005 |
| <60 | -1.27 (4.82) | -1.44 (3.03) | 0.17 [-0.49-0.82] | 0.619 | -0.38 (7.14) | -1.38 (5.11) | 1.00 [-0.19-2.19] | 0.099 |

Legends: In an intention-to-treat (ITT) analysis, patients were followed until October 2021, the end of data availability, or death. In an as-treated (AT) analysis, follow-up was also censored at study-drug discontinuation or comparator-initiation. The risk of the composite kidney outcome (confirmed ≥40% eGFR decline or new end-stage kidney disease) was compared between initiators of GLP-1 RAs versus basal insulin using Cox proportional hazards regression models. Event rates are per 100 patients-years. We calculated an eGFR slope per patient by fitting a linear regression model. We then calculated the mean annual eGFR slope over time for each group and used t-test to compare the treatment groups. eGFR slopes are presented as mL/min/1.73 m^2^.

Abbreviations: GLP-1 RA – Glucagon-like peptide-1 receptor agonists; eGFR –estimated glomerular filtration rate.; ER – event rate.

**Figure S1**: CONSORT diagram describing the formation of the study population.


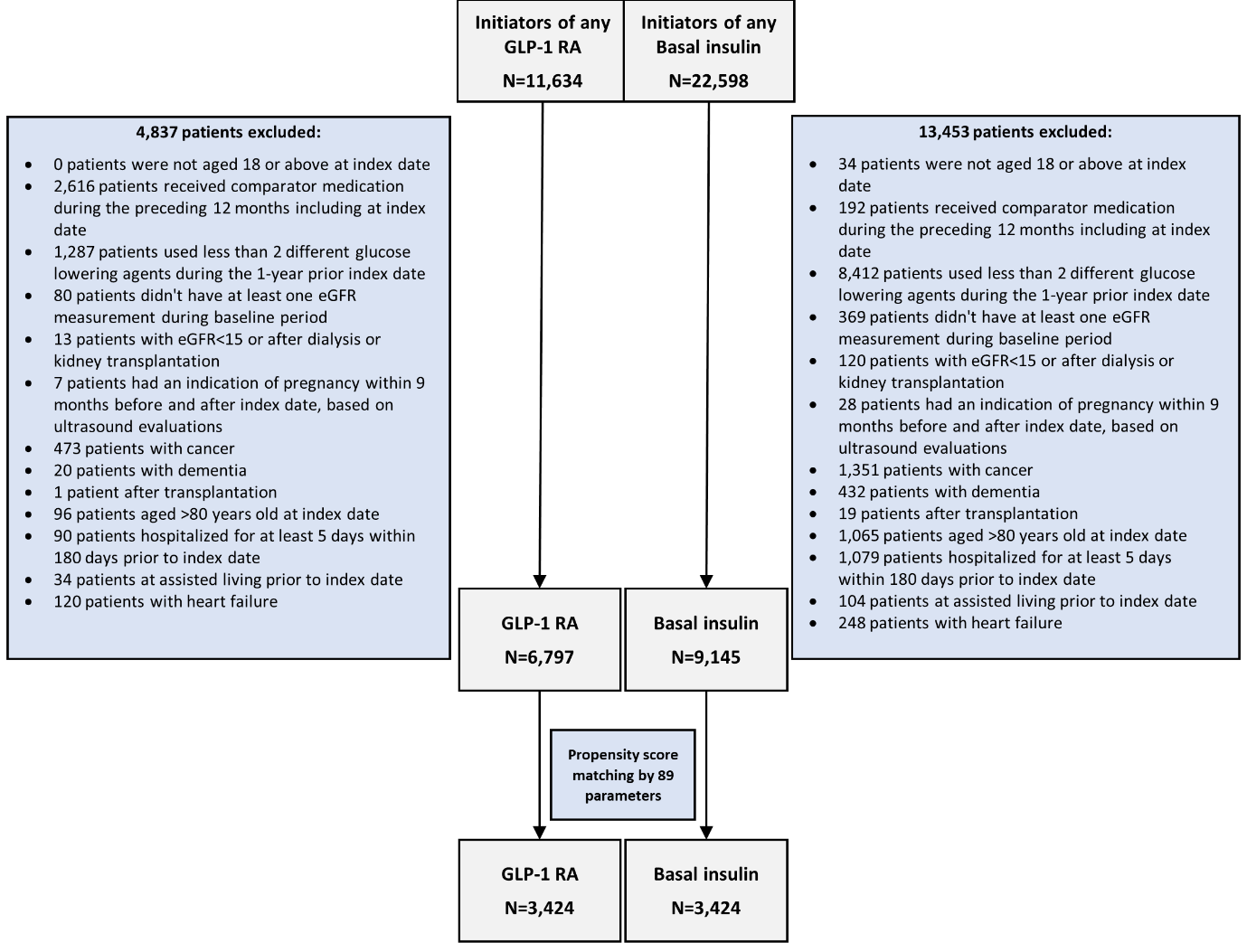


Abbreviations: GLP-1 RA – Glucagon-like peptide-1 receptor agonists; eGFR –estimated glomerular filtration rate

**Figure S2**: The association between initiation of GLP-1 RAs versus basal insulin and the risk of categorical eGFR decline or albuminuria progression in the ITT-48 months analysis.


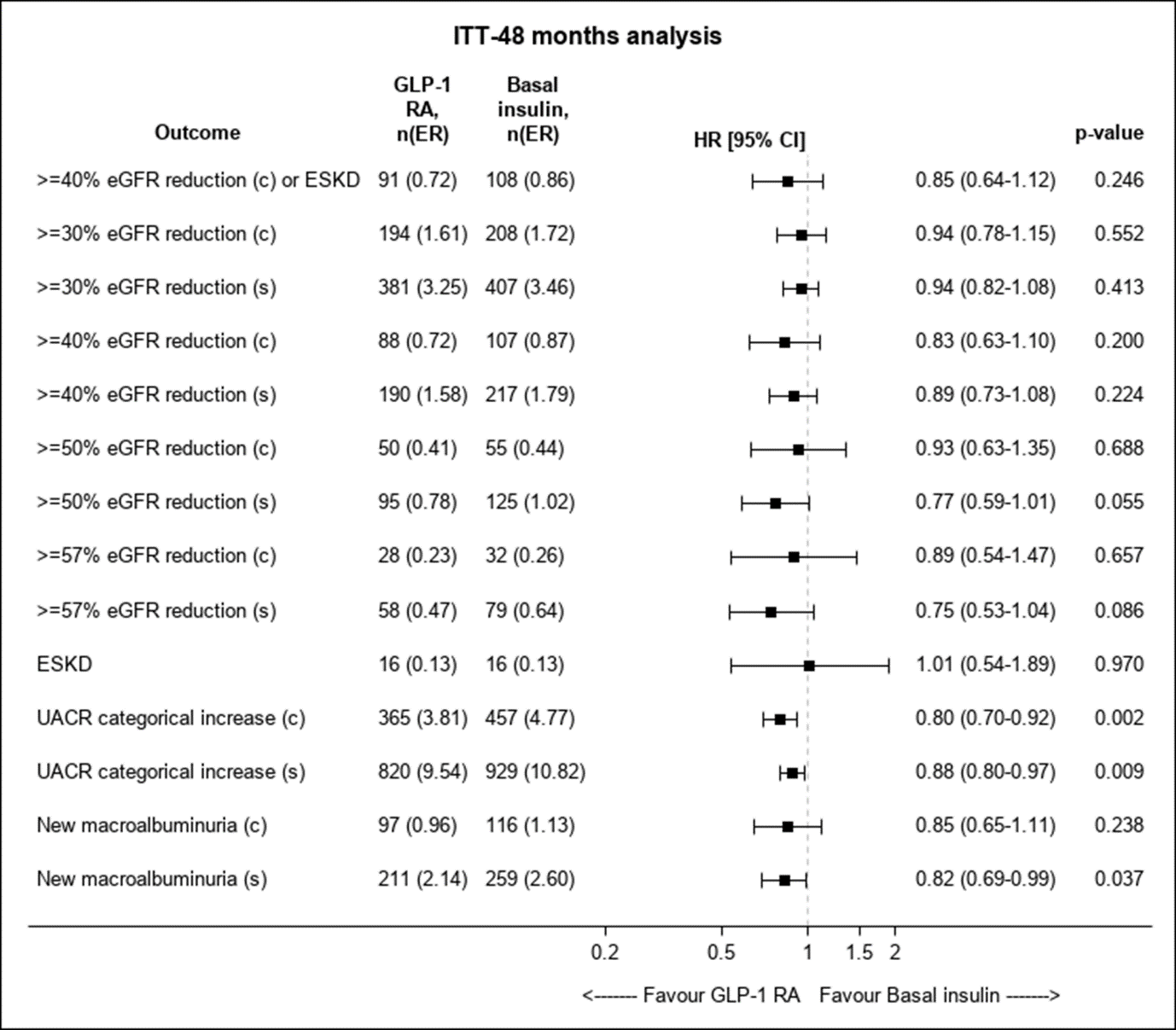


Legends: The risks of GFR decline (by different thresholds) or albuminuria progression were compared between initiators of GLP-1 RA with initiators of basal insulin. In the intention-to-treat (ITT)-48 months analysis, patients were followed until October 2021, end of data availability, death, or until four years of potential follow up. A categorical increase of UACR was defined for the following categories: <30, 30-<300 or ≥300 mg/g. New-onset macroalbuminuria was defined as UACR≥300 among those with UACR≤230 mg/g at baseline (resembling a ≥30% increase in UACR).

Outcomes were assessed as single- (*s*) or confirmed-(*c*) measurements. Cox proportional hazards regression models were applied to compare between treatment arms. Event rate (ER) are presented per 100 patients years.

Abbreviations: GLP-1 RA – Glucagon-like peptide-1 receptor agonists; UACR – Urine albumin-to-creatinine ratio.
